# *Brachymyrmex* species with tumuliform metathoracic spiracles: description of three new species and discussion of dimorphism in the genus (Hymenoptera, Formicidae)

**DOI:** 10.3897/zookeys.371.6568

**Published:** 2014-01-17

**Authors:** Claudia M. Ortiz, Fernando Fernández

**Affiliations:** 1 Instituto de Ciencias Naturales, Universidad Nacional de Colombia, Carrera 30 # 45–03, Bogotá D.C. Colombia

**Keywords:** Brazil, Formicinae, ants, new species, *Brachymyrmex*, dimorphism

## Abstract

*Brachymyrmex* is a taxonomically challenging ant genus that is badly in need of review. Most species are very small and soft bodied and current descriptions regularly lack clarity making species identification a daunting task. Furthermore, the monophyly of *Brachymyrmex* has not been established and the relationships among its species and with closely related genera are poorly understood. Most species of *Brachymyrmex* are monomorphic, but two dimorphic species have been assigned to the genus before. Here, we redescribe these dimorphic taxa, *B. pilipes* and *B. micromegas*, and describe three new monomorphic species, *B. brasiliensis* **sp. n.**, *B. delabiei* **sp. n.** and *B. feitosai* **sp. n.** All five species occur in Brazil and have tumuliform metathoracic spiracles, which are lacking in other *Brachymyrmex* species. We discuss dimorphism and its evolution in the genus and provide a distribution map, illustrations and a species identification key based on workers.

## Introduction

The cosmopolitan ant subfamily Formicinae contains 11 tribes, 60 genera and over 3,000 species, and has been shown to be monophyletic (Shattuck 1992, Johnson et al. 2003, Brady et al. 2000, 2006, Moreau et al. 2006), however, classification within the subfamily is not fully resolved and many genera are in need of thorough review. One of these genera is *Brachymyrmex* Mayr, 1868 which, following the classification proposed by Bolton (2003) belongs to the tribe Plagiolepidini.

*Brachymyrmex* is a genus of minute ants that at first glance exhibit little morphological variation. Currently only the 9-segmented antennae and lack of antennal club have been proposed to diagnose workers of the genus (Bolton 2003). The combination of small size, soft metasoma, and the simple morphology makes observations and interpretation of morphological characters difficult. These difficulties impede taxonomic revisions and even led Creighton (1950) to call *Brachymyrmex* a “miserable little genus”. Nevertheless, 57 described species, subspecies, and varieties are currently assigned to *Brachymyrmex* (Bolton, 2013). The genus has a mainly Neotropical distribution, ranging from the United States to Argentina and Chile, including the Caribbean islands (Kempf 1972, Brandão 1991, Bolton 1995, 2003), but some species have been introduced to Japan (M. Yoshimura, pers. comm.), and Madagascar (Dejean et al. 2010).

The first complete taxonomic revision of *Brachymyrmex* was published by Santschi (1923) and included 27 species and 15 subspecies and varieties. In this revision, Santschi (1923) recognized two subgenera: 1) *Brachymyrmex* *sensu stricto* (including most of the species) and 2) *Bryscha* Santschi, 1923 (four species: *Brachymyrmex (Bryscha) pilipes* Mayr, 1887; *Brachymyrmex (Bryscha) micromegas* Emery in Santschi 1923; *Brachymyrmex (Bryscha) antennatus* Santschi, 1929 and *Brachymyrmex (Bryscha) gaucho* Santschi, 1917). *Brachymyrmex* *sensu stricto* contains species that have hairy legs, antennae without erect hairs and the second segment of the antennal funiculus much shorter that the first (= third antennal segment much shorter than the second). *Bryscha* species have legs and antennae with erect hairs and the second segment of the antennal funiculus is as long as or longer than the first. Unlike other species in the genus, two of the species of the subgenus *Bryscha*, *Brachymyrmex pilipes* and *Brachymyrmex micromegas*, have dimorphic workers. Ambiguity remains regarding the status of *Bryscha*. Brown (1973) provisionally synonymized it under *Brachymyrmex* and Bolton (1995, 2013) accepted this synonymy in his catalogues without substantiating the decision. We tentatively follow these latter authors, but phylogenetic work is required to settle the issue.

Here, as part of a larger taxonomic revision of the genus, we identify and revise the species of *Brachymyrmex* that have tumuliform metathoracic spiracles (= spiracles that are fully dorsal and highly elevated on the meso-metanotum in lateral view). This group contains the two dimorphic *Brachymyrmex* species mentioned above, which we redescribe here. Although tumuliform metathoracic spiracles are not present in any previously known monomorphic *Brachymyrmex* species, we found them present in three new, monomorphic species of the genus, which we describe here. All species with tumuliform metathoracic spiracles occur in Brazil. Hence, the *Brachymyrmex* species of this country may provide new insights into the evolution of dimorphism. We discuss dimorphism in *Brachymyrmex* and the status of the genus.

## Material and methods

### Material and repositories

We studied the *Brachymyrmex* material of the following institutions which includes all relevant types and additional specimens; collection acronyms follow Ward (1989).

CASC California Academy of Sciences, San Francisco, California, USA

CPDC Laboratório de Mirmecologia do Centro de Pesquisas do Cacau, Comissão Executiva do Plano da Lavoura Cacaueira (CEPLAC), Itabuna, Bahia, Brazil

MCZC Museum of Comparative Zoology, Harvard University, Cambridge, USA

MCSN Museo Civico di Storia Naturale “Giacomo Doria”, Genoa, Italy

MZSP Museu de Zoologia da Universidade de São Paulo, São Paulo, Brazil

NHMB Naturhistorisches Museum, Basel, Switzerland

NHMV Naturhistorisches Museum, Wien, Austria

UFUC Universidade Federal de Uberlândia, Uberlândia, Minas Gerais, Brazil

USNM Department of Entomology, National Museum of Natural History Smithsonian Institution, Washington DC, USA

### Images

Photographs of the ants including dorsal, lateral and full-face views of workers and queens were taken at the MCZC with an imaging system that consisted of a Leica MZ16 stereo microscope, a Leica DCF 420 digital camera, and the Auto-Montage Professional software Leica Application Suite 3.7 and Helicon Focus 5.1; and at the USNM with an imaging system that consisted of a Leica Z16APO microscope and a JVC KY-F75U digital camera with a Leica Motor-focus System attached to an IBM IntelM Pro computer, on which composite images were assembled using Auto-Montage Pro Version 5.03.0018 BETA (Synoptics Ltd.). Scanning electron micrographs were taken with a LaB6 electron source. Images were processed with Adobe Photoshop CS. The distribution map was created using the software ArcGIS v10.1 (Esri, Redlands, CA).

### Measurements

Measurements were made using an Advanced Optical Microscope at 120 × magnification and a Leica Z16 APO microscope with a fiber optic ring lamp at 80 × magnification. All measurements are in mm.

*Head Length*_1_ (HL_1_). The maximum length of the head capsule excluding the mandibles; measured in full-face view, as a straight line from the mid-point of the anterior clypeal margin to the mid-point of the posterior (= vertexal) margin of the head (for major workers the posterior margin is defined by a virtual line between the posterior apices of the head).

*Head Length*_2_ (HL_2_). Distance from posterior margin of the frontal triangle (see Bolton 1994, p. 192) to vertexal margin in full-face view.

*Head Length*_3_ (HL_3_). Measurement of the gena in lateral view; this measurement equals the distance from the anterior margin of the eye to the posterior edge of clypeus, perpendicular to this edge.

*Head Width* (HW). The maximum width of the head behind the eyes, measured in full-face view.

*Scape Length* (SL). The maximum length of the scape, excluding the basal constriction that occurs just distal to the condylar bulb.

*Eye Length* (EL). Maximum diameter of the compound eye.

*Weber’s Length* (WL). The diagonal length of the mesosoma, in profile, from the anterior-most point of the pronotum to the posterior-most basal angle of the metapleuron (this measurement excludes the cervical neck of the pronotum).

*Pronotum Length* (PnL). Length from anterior edge to posterior edge of pronotum in dorsal view along the midline (this measurement excludes the cervical neck of the pronotum).

*Pronotum Width* (PnW). Width viewed dorsally, measured from side to side.

*Mesonotum Length* (ML). Length viewed dorsally, measured from anterior edge of mesonotum to mesometanotal suture, with both in the same plane of focus.

*Mesonotum Width* (MW). Width viewed dorsally, measured from side to side.

### Indices

*Cephalic Index* (CI). (HW/HL_1_) × 100.

*Scape Index* (SI_1_). (SL/HW) × 100.

*Scape Index*_1_ (SI_2_). (SL/HL_2_) × 100.

*Ocular Index* (OI). (EL/HW) × 100.

Morphological terminology follows Bolton (1994); terminology for hair inclination follows Kugler (1994).

## Results

### Redescription of dimorphic species

#### Brachymyrmex micromegas

Emery

http://species-id.net/wiki/Brachymyrmex_micromegas

Figs 1–6 ,28

Brachymyrmex (Bryscha) micromegas Emery, in Santschi 1923: 675, figs 30, 32 (w) BRAZIL (MCSN, MZSP) [examined].

##### Lectotype

**(here designated).** 1 minor worker, **Brazil**, São Paulo, Ipiranga (MCSN) [USNM ENT 00757222]. ***Paralectotypes*.** 4 major workers, 1 minor worker, **Brazil**, São Paulo, São Paulo city, Ipiranga (MCSN) [USNM ENT 00757222], (MZSP) [USNM ENT 00757824 – 00757827].

##### Additional material examined.

1 minor worker, **Brazil**, São Paulo, Agudos, 05 Nov 1967, col. W. Kempf (MZSP) [USNM ENT 00757830]; 1 minor worker, **Brazil**, São Paulo, Anhembi, Faz B. Rico, 14 Feb 1969, cols. W. Kempf, J.C. Magalhães, L.T.F.M. Kulman (MZSP) [USNM ENT 00757834].

##### Diagnosis.

**Figure 1–6.**
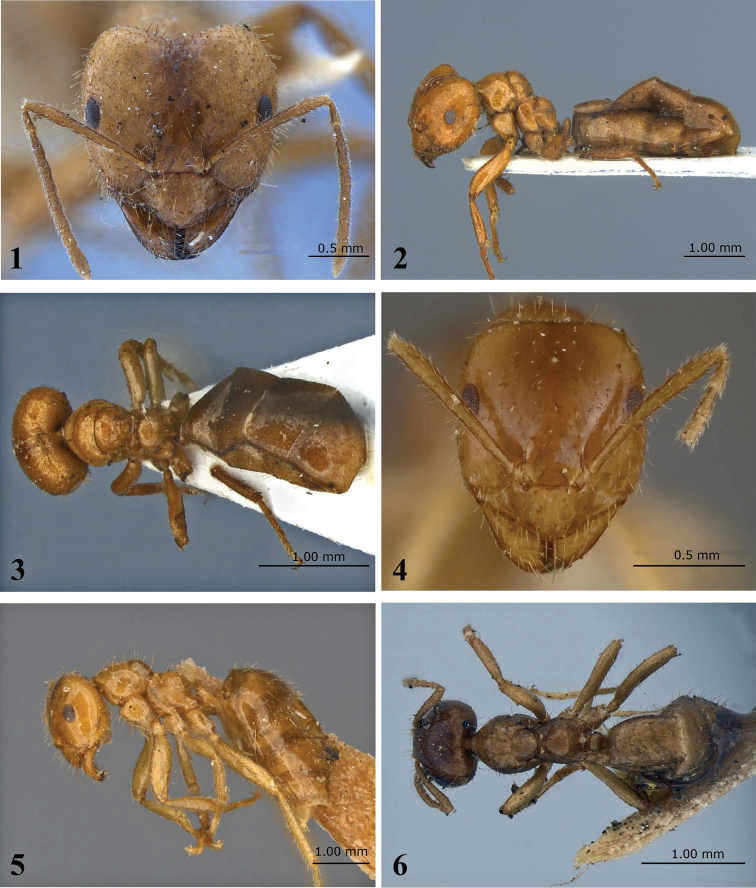
*Brachymyrmex micromegas* Emery (Lectotype) **1** Major worker, head in full-face view **2** Major worker, body in lateral view **3** Major worker, body in dorsal view **4** Minor worker, head in full-face view **5** Minor worker, body in lateral view **6** Minor worker in dorsal view.

This species can be differentiated from most other *Brachymyrmex* species by the following: presence of tumuliform metathoracic spiracles; worker caste dimorphic; toruli touching the posterior clypeal margin, but never surpassing it (best observed in anterodorsal oblique view); and clypeus with a row of long thick hairs near the anterior margin (see Fig. 15). These traits are shared with *Brachymyrmex pilipes*, but *Brachymyrmex micromegas* differs from *Brachymyrmex pilipes* by smooth and shiny body, with very fine longitudinal striations restricted to the metapleura; the body color is usually light brown (Fig. 1).

##### Description

**Minor worker.** *Lectotype measurements* (mm) (n=1) HL_1_ 0.78; HL_2_ 0.49; HL_3_ 0.29; HW 0.70; SL 0.72; EL 0.21; WL 0.98; PnL 0.29; PnW 0.53; ML 0.23; MW 0.53; *Indices* CI 90; SI_1_ 102.77; SI_2_ 148; OI 30.55.

*Paralectotype measurements* (mm) HL_1_ 0.78; HL_2_ 0.49; HL_3_ 0.29; HW 0.70; SL 0.72; EL 0.21; WL 0.98; PnL 0.29; PnW 0.53; ML 0.23; MW 0.53; *Indices* CI 90; SI_1_ 102.77; SI_2_ 148; OI 30.55.

*Additional material examined measurements* (mm) (n=2) HL_1_ 0.83 – 0.94; HL_2_ 0.25 – 0.33; HW 0.77 – 0.86; SL 0.74 – 0.80; EL 0.16 – 0.19; WL 1.0 – 1.10; PnL 0.29 – 0.33; PnW 0.53 – 0.59; ML 0.23 – 0.27; MW 0.25 – 0.33; *Indices* CI 92 – 94; SI_1_ 92 – 97; SI_2_ 71 – 76; OI 21 – 22.

**Description.** Head longer than wide, sub-rectangular. Posterior cephalic border slightly concave. Clypeus large, with rounded anterior margin. Toruli touching the posterior clypeal margin but never surpassing it (best observed in anterodorsal oblique view). Scapes long, surpassing the posterior margin of the head. Ocelli present. Eyes well developed, situated posterior to mid line of head, with 14–15 ommatidia at maximum diameter. Promesonotum in profile conspicuously convex, higher than propodeum. Mesonotum inclined, strongly convex, in profile and separated from pronotum (Fig. 5).

Metanotal groove deep, wide. Metathoracic spiracles fully dorsal, tumuliform, bulging out of the metanotal groove in lateral view (Fig. 5). Propodeal spiracle near to declivity of propodeum. Petiolar scale not inclined forward.

Body smooth and shiny, except for the metapleura, which have very fine, longitudinal striations. Most of mandibular surface smooth and shiny (best observed in anterodorsal oblique view). Entire body, including antennae, legs and palps with conspicuous erect and sub-erect pilosity. Clypeus with a row of many long thick hairs near the anterior margin, the rest of the clypeal surface with many shorter hairs. Gaster smooth and shiny without scattered long erect hairs and without dense pubescence. Body usually light brown, gaster often darker.

**Major worker.** *Paralectotype measurements* (mm) (n=3). HL_1_ 1.66 – 1.88; HL_2_ 1.17; HL_3_ 0.57 – 0.63; HW 1.66 – 1.83; SL 1.12 – 1.23; EL 0.25 – 0.28; WL 1.66 – 1.8; PnL 0.49 – 0.73; PnW 1.05 – 1.13; ML 0.55 – 0.63; MW 0.63 – 0.75; *Indices* CI 95 – 100; SI_1_ 61.6 – 74.12; SI_2_ 105; OI 15.1 – 15.7.

**Description.** Head strongly cordate, broader at eye level (Fig. 1). Clypeus large, with anterior margin rounded. Toruli touching the posterior clypeal margin but never surpassing it. Scapes surpassing the posterior margin of the head. Ocelli present. Eyes well developed, situated posterior to the midline of the head. Promesonotum in profile strongly convex, higher than the propodeum.

Metanotal groove deep. Metathoracic spiracles fully dorsal, tumuliform, bulging out of the metanotal groove in lateral view (Fig. 2). Propodeal spiracle near to declivity of propodeum. Petiole scale not inclined forward, rounded.

Body smooth and shiny, except for the metapleura, which has very fine, longitudinal striations. The entire body, including antennae, legs and palps with conspicuous, erect and sub-erect pilosity. Clypeus with a row of many long thick hairs near anterior margin, the rest of the clypeal surface shiny and with many shorter hairs. Body light brown.

**Queen and male.** Unknown.

##### Distribution.

Brazil: São Paulo State.

##### Remarks.

Because the specimens were poorly mounted, not all measurements could be taken on all ants. The type-series was collected near the locality where the Independência Park arboretum is now located, just beside the MZSP building in Ipiranga, São Paulo, Brazil. Despite recent field trips to the locality, none specimens have been collected (R.M. Feitosa, pers. comm.).

#### Brachymyrmex pilipes

Mayr

http://species-id.net/wiki/Brachymyrmex_pilipes

Figs 7 –16 ,28

Brachymyrmex pilipes Mayr, 1887: 524 (q.m.) BRAZIL (NHMV) [examined]. Santschi 1929: 310 (w.). BRAZIL (NHMB) [examined]. Combination in *Brachymyrmex (Bryscha)*: Santschi 1923: 674.

##### Lectotype

**(here designated).** 1 queen, ***Paralectotypes*** 1 queen, 1 male, **Brazil**, Santa Catharina (NHMV).

##### Additional material examined.

6 minor workers, **Brazil**, Paraná, Rio Azul, 1000 m, Oct. 1959, col. F. Plaumann (MZSP) [USNM ENT 00757822, USNM ENT 00757823]; 2 major workers, 2 minor workers, 1 queen, **Brazil**, Paraná, Río Negro (NHMB); 3 minor workers, **Brazil**, Santa Catharina, Chapecó, Dic. 1957, col. F. Plaumann leg. (MZSP) [USNM ENT 00757821]; 6 minor workers, **Brazil**, Santa Catharina, Nova Teutonia, 27°11'S, 52°23'W, 300–500 m, Jul.1958, col. F. Plaumann (MZSP) [USNM ENT 00757829, USNM ENT 00757831, USNM ENT 00757832]; 1 minor worker, **Brazil**, Río de Janeiro, Nova Friburgo, Fazenda Barreto, 22°9'40.4712"S, 42°31'27.4866"W, 1068 m, 11–12 Jun 2011, col. T.M.S. Mesquita (UFUC) [USNM ENT 00757824]; 1 minor worker, **Brazil**, São Paulo, Ubatuba, Parque Estadual da Serra do Mar, Núcleo Santa Virgínia, 23°19'S, 45°06'W, 870–1000 m, 22 Apr 2005, col. M. Uehara (MZSP) [USNM ENT 00757823].

##### Diagnosis.

This species can be differentiated from most of the *Brachymyrmex* by the following: presence of tumuliform metathoracic spiracles; worker caste dimorphic; toruli touching the posterior clypeal margin, but never surpassing it (best observed in anterodorsal oblique view); and long thick hairs in a row near anterior clypeal margin (Fig. 15). These traits are shared with *Brachymyrmex micromegas*, but *Brachymyrmex pilipes* differs by the fine, longitudinal striations on most of the mesosoma (Fig. 16), and the gaster color often is darker than the body color.

**Figure 7–12.**
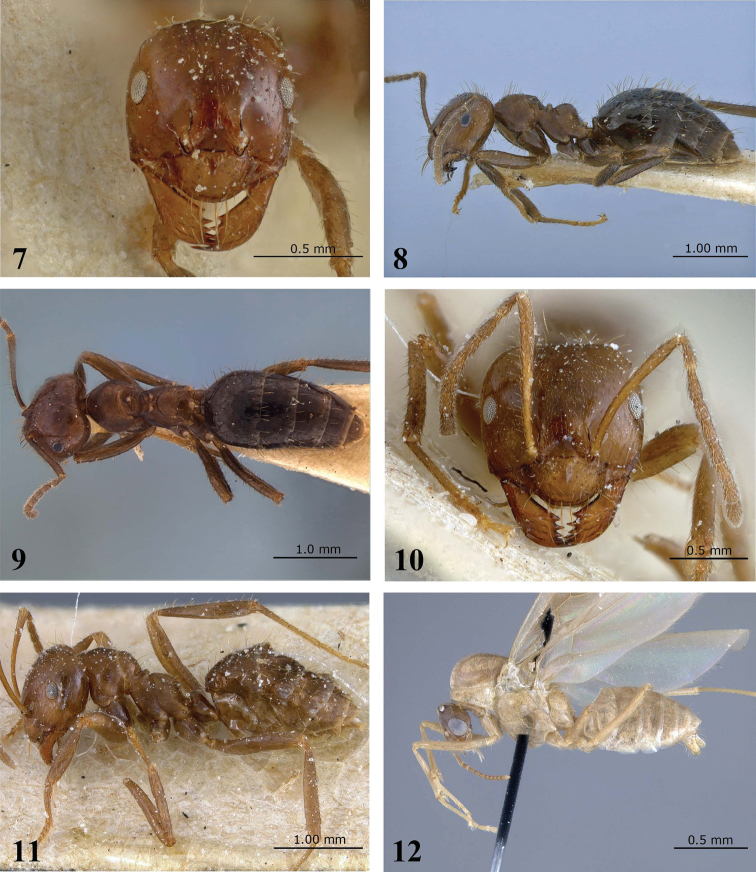
*Brachymyrmex pilipes* Mayr **7** Minor worker, head in full-face view **8** Minor worker, body in lateral view **9** Minor worker, body in dorsal view **10** Major worker, head in full-face view **11** Major worker, body in lateral view **12** Male, body in lateral view.

**Figure 13–16.**
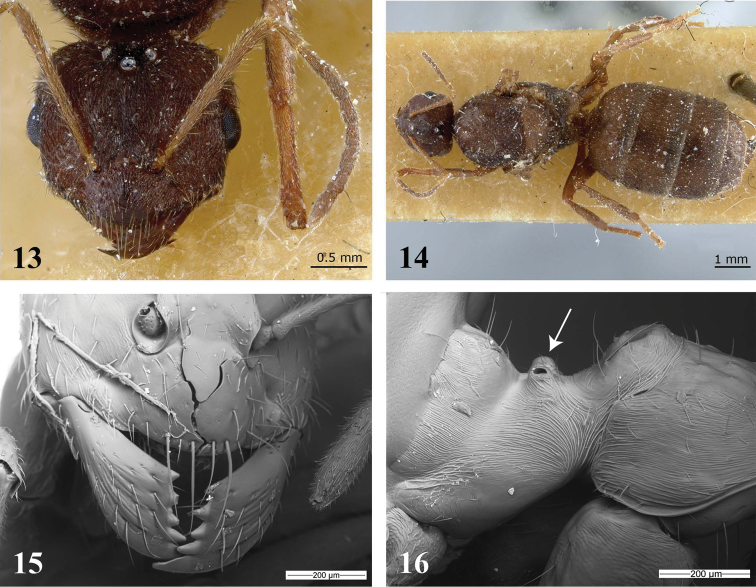
*Brachymyrmex pilipes* Mayr. **13** Queen (Lectotype), head in full-face view **14** Queen (Lectotype), body in dorsal view **15** Minor worker, clypeus **16** Minor worker, mesosoma in right lateral view.

##### Description

**Minor worker.** *Measurements* (mm) (n=8) HL_1_ 0.62 – 1.21; HL_2_ 0.50 – 0.74; HL_3_ 0.33 – 0.39; HW 0.60 – 1.07; SL 0.57 – 1.17; EL 0.12 – 0.22; WL 0.97 – 1.42; PnL 0.40 – 0.55; PnW 0.64 – 0.76; ML 0.20 – 0.33; MW 0.33 – 0.37; *Indices* CI 88 – 97; SI_1_95 – 109; SI_2_114 – 158; OI 18 – 20.

**Description.** Head slightly longer than wide, almost squared, slightly narrowing anteriorly. Posterior cephalic border slightly concave. Clypeus large, with rounded anterior margin. Toruli touching the posterior clypeal margin, but never surpassing it (best observed in anterodorsal oblique view) (Fig. 15). Scapes long, surpassing the posterior margin of the head. Ocelli present. Eyes well developed, located at the midline of the head, with 10–11 ommatidia at their maximum diameter. Mesonotum conspicuously convex. Metanotal groove deep and wide. Metathoracic spiracles fully dorsal, tumuliform, bulging out of the metanotal groove in lateral view (Fig. 8). Propodeum strongly convex, unarmed. Propodeal spiracle near to posteriopropodeal margin. Petiole scale rounded and not inclined forward.

Head and gaster smooth and shiny. Mesosoma with very fine, dense longitudinal and oblique striations. Most of mandibular surface with longitudinal rugulae (best observed in anterodorsal oblique view). Entire body, including antennae, legs, and palps with conspicuous erect and suberect pilosity that are larger on dorsum. Long thick hairs in a row near to the anterior clypeal margin. Most of clypeal surface with many erect hairs, which are shorter than the thick hairs. Body light brown, gaster dark brown, hairs lighter.

**Major worker.** *Measurements* (mm) (n=2) HL_1_ 1.44 – 1.46; HL_2_ 0.92 – 0.94; HL_3_ 0.37 – 0.39; HW 1.35; SL 1.17; EL 0.20 – 0.21; WL 1.60 – 1.83; PnL 0.59; PnW 0.88; ML 0.39; MW 0.49. *Indices* CI 92; SI_1_ 104.34; SI_2_ 58 – 68; OI 17.39.

**Description.** Head bigger than that of the minor worker, squared with posterior corners angulate and posterior cephalic border slightly concave. Clypeus large, with rounded anterior margin. Toruli touching the posterior clypeal margin, never surpassing it. Scapes long, surpassing the posterior margin of the head. Ocelli present. Eyes well developed, located at mid line of head, with 11 ommatidia at their maximum diameter.

Mesonotum conspicuously convex. Metanotal groove present. Metathoracic spiracles fully dorsal, tumuliform, bulging out of the metanotal groove in lateral view (Fig. 11). Propodeum strongly convex, unarmed. Propodeal spiracle near posteriopropodeal margin. Petiolar scale rounded and inclined forward.

Head and gaster smooth and shiny. Mesosoma with very fine, dense longitudinal and oblique striations. Entire body, including antennae, legs, and palps with conspicuous erect and suberect pilosity that is longer on the dorsum. Long hairs in a row near anterior clypeal margin. Most of clypeal surface with abundant erect hairs, which are shorter than the thick hairs. Body light brown, gaster dark brown, hairs lighter colored.

**Queen.** *Lectotype measurement* (mm) (n=1) HL_1_ 1.61; HL_2_ 0.42; HW 1.88; SL 1.54; EL 0.47; WL 4.04; PnL 2.21; PnW 2.19; ML 0.97; MW 1.59. *Indices* CI 116.17; SI_1_ 82.27; OI 25.31.

**Description.** Same as worker except for standard queen modifications and the following: abundant erect hairs and dense pubescence on entire body. There is a row of thick hairs near the anterior margin of clypeus, similar to the workers and abundant pubescence on the body (Fig. 13). Body dark brown.

**Male.** *Paralectotype measurements* (mm) (n=1) HL 0.31; EL 0.16; WL 0.88.

**Description.** Scapes surpassing the posterior margin of the head, few erect hairs, and sparse pubescence on the body; some sparse long hairs on the tibiae. Head brown, mesosoma and gaster yellow. Penis valves longer than parameres *in situ*.

##### Distribution.

Brazil: states of Paraná, Santa Catharina, São Paulo and Rio de Janeiro.

##### Remarks.

The specimen from São Paulo at Ubatuba, Parque Estadual da Serra do Mar, Núcleo Santa Virgínia [USNM ENT 00757823] was collected in an area of relatively well-preserved mature forest with some remnants of primary forest where selective logging took place until 1970 (Magrini et al. 2011). Habitat information is lacking for the other specimens.

### Description of new monomorphic species

#### Brachymyrmex brasiliensis sp. n.

http://zoobank.org/84634C4B-8171-4A8A-B851-E9296D863F3B

http://species-id.net/wiki/Brachymyrmex_brasiliensis

Figs 19–21 ,28

##### Holotype worker

(MZSP) [USNM 00757748] and ***Paratype*** *worker* (UFUC) [USNM ENT 00757833]: **Brazil**, Rio de Janeiro, Nova Friburgo, Fazenda Barreto, 22°9'40.4712"S, 42°31'27.4866"W, 1068 m, 11–12 Jun 2011, col. T.M.S. Mesquita.

##### Additional material examined.

1 worker, **Brazil**, Goiás, Anápolis, 12 Feb 1958, col. W. Kempf (MZSP) [USNM ENT 00757820].

##### Diagnosis.

**Figure 17–21.**
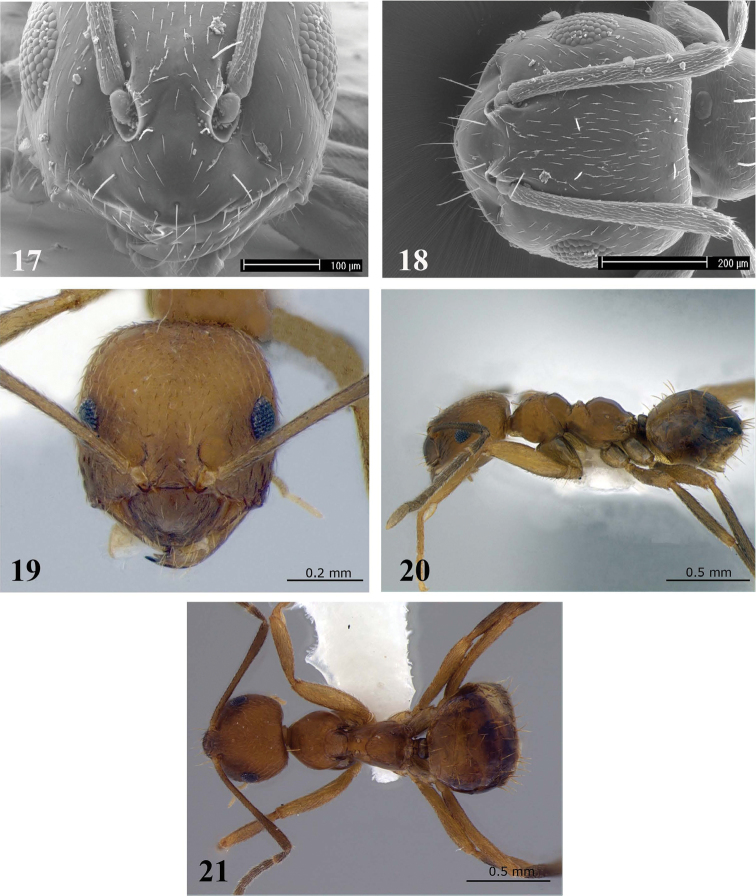
**17** Frontal view of the clypeus of a *Brachymyrmex* monomorphic specimen showing the distribution of hears: one central hair near to the anterior margin; one pair of lateral hairs at clypeus midlength and one pair of hairs near the toruli **18** Head of the same specimen in semidorsal view (SEM pictures, were taken by J. Cillis, T. Delsinne and M. Leponce (RBINS)). *Brachymyrmex brasiliensis* sp. n. (Holotype) **19** Worker, head in full-face view **20** Worker, body in lateral view **21** Worker, body in dorsal view.

This species differs from most other *Brachymyrmex* species by the presence of tumuliform metathoracic spiracles, and clypeus with five long, erect hairs arranged as follows: one central hair near to the anterior margin, usually conspicuous; one pair of lateral hairs at clypeus midlength and one pair of hairs near the toruli (see Fig. 17–18). Unique features for *Brachymyrmex brasiliensis* are the smooth and shiny gaster and, opaque head and mesosoma.

##### Description

**Worker.** *Holotype measurements* (mm) HL_1_ 0.59; HL_2_ 0.36; HL_3_ 0.15; HW 0.53; SL 0.67; EL 0.11; WL 0.71; PnL 0.24; PnW 0.36; ML 0.15; MW 0.18; *Indices* CI 90; SI_1_ 127; SI_2_187.5; OI 25.

*Paratype measurements* (mm) HL_1_ 0.59; HL_2_ 0.36; HL_3_ 0.15; HW 0.53; SL 0.67; EL 0.11; WL 0.71; PnL 0.24; PnW 0.36; ML 0.15; MW 0.18; *Indices* CI 90; SI_1_ 127; SI_2_187.5; OI 25.

*Additional material examined* (mm) HL_1_ 0.61; HL_2_ 0.40; HL_3_ 0.15; HW 0.53; SL 0.68; EL 0.13; WL 0.73; PnL 0.24; PnW 0.36; ML 0.15; MW 0.18; *Indices* CI 89; SI_1_ 129; SI_2_ 187.5; OI 25.

**Description.** Head slightly longer than wide. Posterior cephalic border slightly convex, sides slightly convex. Anterior clypeal margin rounded. Toruli touching the posterior clypeal margin but never surpassing it (best observed in anterodorsal oblique view; see Fig. 17). Scapes long, surpassing the posterior margin of the head. Ocelli present. Eyes located at cephalic midline and well developed with 11 ommatidia at their maximum diameter.

Promesonotum convex in profile, mesonotum strongly convex and separated from pronotum. Propodeum convex with short dorsum. Metathoracic spiracles fully dorsal, tumuliform, bulging out of the metanotal groove in lateral view. Propodeal spiracle round, separated from declivity of propodeum by a distance equal to the diameter. Petiole short, apex rounded and inclined forward.

Head and mesosoma finely punctate and opaque. Dorsum of head with some erect hairs on front and with scattered decumbent pubescence. Gaster smooth and shiny with several scattered erect hairs. Scapes with decumbent hairs that are shorter than the maximum scape diameter. Clypeus with five long, erect hairs arranged as follows: one central hair near to the anterior margin, usually conspicuous; one pair of lateral hairs at clypeus midlength and one pair of hairs near the toruli. Pronotum usually with two erect hairs, rest of mesosoma without hairs. Body light brown.

**Queen and male.** Unknown.

##### Etymology.

After Brazil, the country of collection, in honor of its very rich ant fauna.

##### Distribution.

Brazil: states of Goiás and Rio de Janeiro.

##### Remarks.

Biological and ecological information of this species is lacking.

#### Brachymyrmex delabiei sp. n.

http://zoobank.org/17FF8984-4DCA-48B2-BC39-0DCA9B702B8A

http://species-id.net/wiki/Brachymyrmex_delabiei

Figs 22–24 ,28

##### Holotype worker

(MZSP) [USNM ENT 00757718] and ***Paratypes*** 3 workers (CPDC [USNM ENT 00757719], ICN [USNM ENT 00757720], USNM [USNM ENT 00757721]): **Brazil**, São Paulo, Tapiraí, 24°01'55.5"S, 47°27'56"W, 08–14 Jan 2001, col. R.R. Silva & Eberhardt, Transecto 1 Winkler 23.

##### Additional material examined.

1 worker, **Brazil**, Bahia, Boa Nova, João Mata, 13 Aug 2003, cols. J.R.M. Santos & J.C.S. Carmo (CPDC) [USNM ENT 00757610]. 1 worker, **Brazil**, Bahia A61 Camacan, 27 Aug 1999. 15°36'04"S, 39°31'16"W, col. J.R.M. dos Santos, (CPDC) [USNM ENT 00757837]; 1 worker, Brazil, Santa Catharina, Palhoça, PE Serra do Tabuleiro, 02–10 Nov 2003, 27°44'28"S, 48°41'50"W, cols. R.R. Silva, B.H. Dietz and A. Tavares, (MZSP) [USNM ENT 00757725]; 1 worker, **Brazil**, São Paulo, São Bernardo do Campo, 01 Jun 1971, cols. W.L. & D.E. Brown (MCZC) [USNM ENT 00757835].

##### Diagnosis.

This species differs from most other *Brachymyrmex* species by the presence of tumuliform metathoracic spiracles, and clypeus with five long, erect hairs arranged as follows: one central hair near to the anterior margin, usually conspicuous; one pair of lateral hairs at clypeus midlength and one pair of hairs near the toruli (see Fig. 17–18) and from *Brachymyrmex brasiliensis* by its entirely smooth and shiny body. *Brachymyrmex delabiei* can be diagnosed from *Brachymyrmex feitosai* sp. n. (see below) by the lack of dense pubescence on the first segment of the gaster and by the presence of erect hairs on the mesosoma; two on the pronotum and two on the mesonotum.

**Figure 22–27.**
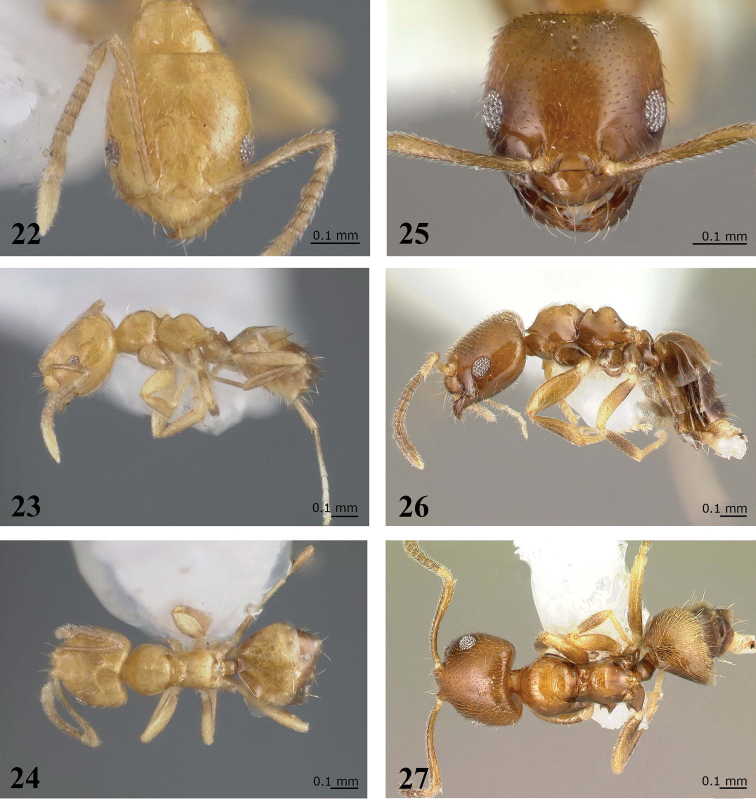
*Brachymyrmex delabiei* sp. n. (Holotype). **22** Worker, head in full-face view **23** Worker, body in lateral view **24** Worker, body in dorsal view. *Brachymyrmex feitosai* sp. n. (Paratype) **25** Worker, head in full-face view **26** Worker, body in lateral view **27** Worker, body in dorsal view (pictures taken by http://www.antweb.org/).

**Figure 28.**
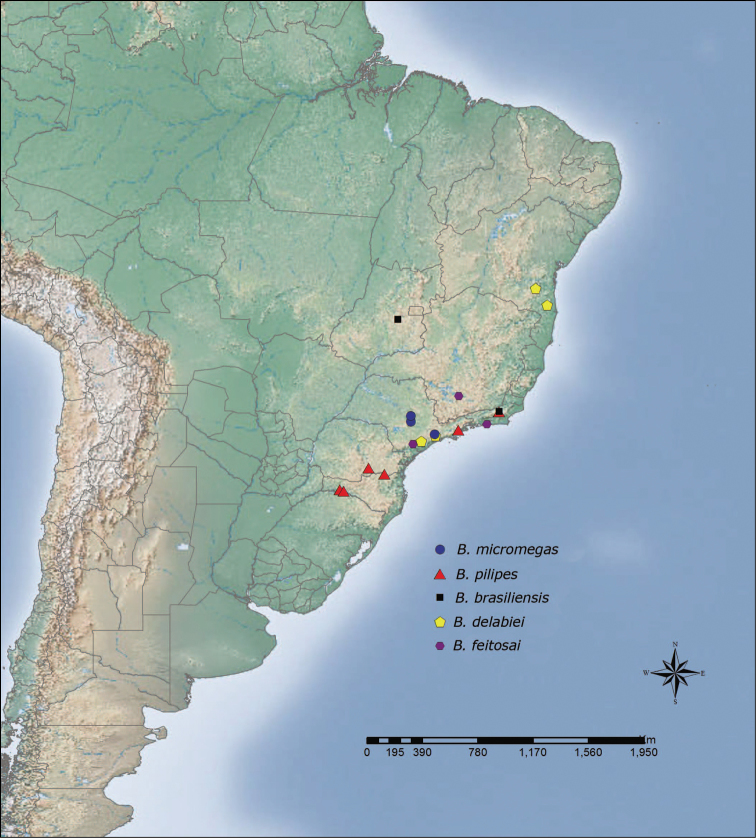
Distribution map of *Brachymyrmex micromegas*, *Brachymyrmex pilipes*, *Brachymyrmex brasiliensis* sp. n., *Brachymyrmex delabiei* sp. n., *Brachymyrmex feitosai* sp. n.

##### Description

**Worker.** *Holotype measurements* (mm). HL_1_ 0.38; HL_2_ 0.29; HL_3_ 0.08; HW 0.32; SL 0.32; EL 0.07; WL 0.40; PnL 0.12; PnW 0.22; ML 0.07; MW 0.13; *Indices* CI 83.7; SI_1_ 102.8; SI_2_134.4; OI 22.2.

*Paratypes measurements* (mm) (n=3). HL_1_ 0.34 – 0.41; HL_2_ 0.22 – 0.30; HL_3_ 0.06 – 0.09; SL 0.31 – 0.36; EL 0.07 – 0.09; WL 0.35 – 0.43; PnL 0.09 – 0.13; PnW 0.15 – 0.24; ML 0.07 – 0.12; MW 0.13 – 0.17; *Indices* CI 83.7 – 93; SI 97 – 119; SI_2_112 – 135; OI 21.05 – 26.

*Additional material examined measurements* (mm) (n=4). HL_1_ 0.34 – 0.41; HL_2_ 0.22 – 0.28; HL_3_ 0.06 – 0.09; SL 0.31 – 0.36; EL 0.08 – 0.09; WL 0.30 – 0.43; PnL 0.10 – 0.12; PnW 0.15 – 0.20; ML 0.07 – 0.12; MW 0.15; *Indices* CI 88.8 – 92; SI_1_ 97 – 100; SI_2_71 – 78; OI 25.

**Description.** Head slightly longer than wide, sides slightly convex. Posterior cephalic border flat, slightly concave in the middle. Anterior clypeal margin rounded. Toruli surpassing the posterior clypeal margin (best observed in anterodorsal oblique view). Scapes surpassing the posterior margin of the head. Ocelli present. Eyes situated below the cephalic midline and well developed with 7–9 ommatidia at their maximum diameter.

Promesonotum convex in profile. Mesonotum strongly convex, rounded and separated from pronotum. Propodeum strongly convex with short dorsum. Metathoracic spiracles fully dorsal, tumuliform, bulging out of the metanotal groove in lateral view, equidistant in diameter from metanotal groove and from the propodeal folding. Propodeal spiracle round, elevated from integument on the propodeal border. Petiole short and inclined forward.

Body smooth and shiny. Dorsum of head, promesonotum, and propodeum with short appressed hairs. Scapes with suberect and subdecumbent hairs. Clypeus with five long, erect hairs arranged as follows: one central hair near to the anterior margin, usually conspicuous; one pair of lateral hairs at clypeus midlength and one pair of hairs near the toruli. Gaster with several scattered long erect hairs, without dense pubescence. Mesosoma with erect hairs, two on the pronotum and two on the mesonotum. Body yellowish.

**Queen and male.** Unknown.

##### Distribution.

Brazil: states of Bahia, Santa Catharina and São Paulo.

##### Etymology.

We are pleased to name this ant in honor of Dr Jacques Delabie (CPDC) for his contribution to ant taxonomy and biology and his unconditional support for many ant biologists working in the Neotropics.

##### Remarks.

The type specimens from São Paulo at Tapiraí [USNM ENT 00757718 – 00757722] were collected in a pristine region of the Brazilian Atlantic Forest (R.M. Feitosa, pers. comm.). Habitat information is lacking for the other specimens.

#### Brachymyrmex feitosai sp. n.

http://zoobank.org/01B69600-0156-4B6E-B162-700C921FDFBD

http://species-id.net/wiki/Brachymyrmex_feitosai

Figs 25 –28

##### Holotype and paratypes.

2 workers (MZSP) [USNM ENT 00757694]: **Brazil**, Rio de Janeiro, Floresta de Tijuca, D. Federal. 16 Dec 1959, C.A: Campos Seabra.

##### Additional material examined.

3 workers **Brazil**, Minas Gerais, Lavras, Ijaci e Perdões, 21°00'–21°19'S, 44°00'–45°07'W, Fragmento, 06 à 12/2002, cols. M.S. Santos & N.S. Dias (CPDC) [USNM ENT 00757836]; 1 worker, **Brazil**, São Paulo, Sete Barras, PE Carlos Botelho, 600 m, 24°12'02"S, 47°58'43"W. 11–15 May 2009, armadilha subterrânea #18, F. Esteves et al. cols. (MZSP) [ANTWEB CASENT 0217326].

##### Diagnosis.

This species differs from most other *Brachymyrmex* species by the presence of tumuliform metathoracic spiracles, and clypeus with five long, erect hairs arranged as follows: one central hair near to the anterior margin, usually conspicuous; one pair of lateral hairs at clypeus midlength and one pair of hairs near the toruli (see Fig. 17–18). Like *Brachymyrmex delabiei* it differs from *Brachymyrmex brasiliensis* by its entirely smooth and shiny body. *Brachymyrmex feitosai* can be diagnosed from *Brachymyrmex delabiei* by the dense pubescence on the first gastral segment and by the presence of many suberect hairs on the pronotum and mesonotum.

##### Description

**Worker.** *Holotype measurements* (mm). HL_1_ 0.40; HL_2_0.28; HL_3_ 0.09; HW 0.36; SL 0.31; EL 0.10; WL 0.45; PnL 0.18; PnW 0.28; ML 0.09; MW 0.18; *Indices* CI 88.8; SI_1_ 87.5; SI_2_ 88.6; OI 27.5.

*Paratypes measurements* (mm) (n=2) HL_1_ 0.33 – 0.43; HL_2_ 0.27 – 0.29; HL_3_ 0.07 – 0.10; HW 0.29 – 0.39; SL 0.26 – 0.34; EL 0.06 – 0.10; WL 0.33 – 0.45; PnL 0.08 – 0.15; PnW 0.20 – 0.24; ML 0.05 – 0.11; MW 0.13 – 0.17; *Indices* CI 82 – 90; SI_1_ 88 – 103; SI_2_105 – 113; OI 22 – 29.

*Additional material examined* (mm) (n=3) HL_1_ 0.40 – 0.43; HL_2_0.27 – 0.29; HL_3_ 0.09; HW 0.35 – 0.39; SL 0.31; EL 0.10; WL 0.42 – 0.45; PnL 0.09 – 0.18; PnW 0.25 – 0.28; ML 0.08 – 0.12; MW 0.17 – 0.19; *Indices* CI 88 – 89; SI_1_ 81 – 88; SI_2_88 – 94; OI 25 – 28.

**Description.** Head slightly longer than wide. Posterior cephalic border slightly concave in the middle, and sides slightly convex. Clypeus with rounded anterior margin. Toruli surpassing the posterior clypeal margin (best observed in anterodorsal oblique view). Scapes reaching posterior margin of the head, but not surpassing it. Ocelli present. Eyes located below cephalic midline and well developed with 7–8 ommatidia at their maximum diameter.

Promesonotum convex in profile, mesonotum strongly convex and separated from pronotum. Metanotal groove present. Metathoracic spiracles dorsal, strongly protruding, fully dorsal, tumuliform, bulging out of the metanotal groove in lateral view, equidistant from the metanotal groove and the propodeal fold. Propodeum strongly convex with short dorsum. Propodeal spiracle round, elevated from integument on the propodeal border.

Body smooth and shiny. Petiole short, apex rounded and inclined forward. Scapes with suberect hairs. Clypeus with five long, erect hairs arranged as follows: one central hair near to the anterior margin, usually conspicuous; one pair of lateral hairs at clypeus midlength and one pair of hairs near to toruli. Dorsum of head, promesonotum and propodeum with conspicuous semi-erect hairs lighter than body color. Gaster with several scattered long erect hairs and with dense pubescence on the first gastral segment that is lighter in color. Body brown.

**Queen and male.** Unknown.

##### Distribution.

Brazil: states of Minas Gerais, Rio de Janeiro and, São Paulo.

##### Etymology.

We are pleased to name this ant in honor of our friend and colleague, Dr Rodrigo Feitosa (Universidade Federal do Paraná) for his great contributions to ant taxonomy and his unconditional support for taxonomists, young and old.

##### Remarks.

The specimen from São Paulo at Sete Barras [ANTWEB CASENT 0217326] was collected in a pristine region of the Brazilian Atlantic Forest. It was obtained in an underground trap, possibly indicating this species has hypogaeic habits (R.M. Feitosa, pers. comm.). Habitat information is lacking for the other specimens.

### Key to workers for the *Brachymyrmex* species with tumuliform metathoracic spiracles

**Table:** 

1	Clypeus with a single long apical hair near to the anterior margin, two lateral hairs medially and two hairs near the toruli (Figs 17–18); monomorphic	2
–	Clypeus with a continuous row of long thick hairs near the anterior margin (Fig. 15), and remaining pilosity not arranged as above; dimorphic	4
2(1)	Toruli surpassing the posterior clypeal margin (best observed in anterodorsal oblique view) (Fig. 17); entire body smooth and shiny	3
–	Toruli touching the posterior clypeal margin but never surpassing it (best observed in anterodorsal oblique view); head and mesosoma finely punctate and opaque; gaster smooth and shiny (Figs 20–21)	*Brachymyrmex brasiliensis*
3(2)	Mesosoma without erect hairs (Fig. 26); first gastral segment with dense yellowish pubescence	*Brachymyrmex feitosai*
–	Mesosoma with two erect hairs on pronotum and two on mesonotum (Fig. 23); gaster without pubescence (Fig. 24)	*Brachymyrmex delabiei*
4(1)	Mesosoma mostly smooth and shiny, except for longitudinal striations restricted to the metapleura; body entirely light brown	*Brachymyrmex micromegas*
–	Mesosoma with fine longitudinal striations all over (Fig. 16); gaster darker than the rest of body	*Brachymyrmex pilipes*

## Discussion

*Brachymyrmex* was classified as being an entirely monomorphic genus by Bolton (2003), even though Santschi (1923) described two dimorphic species almost a century ago. Quirán et al. (2004) indicated that *Brachymyrmex* is a dimorphic genus but these authors do not treat dimorphic species in their paper, and neither do subsequent studies on *Brachymyrmex* from Argentina or elsewhere. Here, we redescribed the dimorphic *Brachymyrmex* species and we confirm that, as currently defined, *Brachymyrmex* should be added to the list of genera with monomorphic and dimorphic workers.

Moreover, while studying museum material we identified a number of specimens of described and new monomorphic *Brachymyrmex* species not dealt with here that have intercastes (a queen-worker intermediate) (Ortiz, pers. obs.). These observations, though preliminary, raise questions about the evolution of castes in Formicinae. Some authors (Wilson 1953, Hölldobler and Wilson 1990) suggested that caste evolution may occur through disruptive selection on allometric differences among the workers and these authors used allometry to classify specimens to castes. Baroni Urbani and Passera (1996) proposed that soldiers evolved independently of workers, and that they originated directly from the queen, but this proposal was refuted by Ward (1997). Recently, Molet et al. (2012) suggested that novel castes such as soldiers and ergatoid queens evolve from rare intercastes, which consist of anomalous specimens with characteristics of both winged queens and workers. These ‘developmental mosaics’ would be erratically produced by colonies experiencing environmental or genetic perturbations; as earlier suggested by Wheeler (1991), and Anderson et al. (2008). The debate on the evolution of castes in ants is not fully settled. If *Brachymyrmex* truly contains both monomorphic and dimorphic species, the genus would provide an interesting system for studying competing hypotheses on the origin of castes in a phylogenetic framework.

One could question whether these two dimorphic species should be assigned to *Brachymyrmex* given the substantial morphological differences we observed: dimorphic species are considerably larger than the monomorphic ones and they have a long row of thick hairs near the anterior clypeal margin (Fig. 15). This contrasts sharply with the character states observed in the monomorphic species, all of which have a clypeus with five long, erect hairs arranged as follows: one central hair near to the anterior margin, usually conspicuous; one pair of lateral hairs at clypeus midlength and one pair of hairs near the toruli (Figs 17–18). Hence, the only taxonomic traits that tie these dimorphic species to *Brachymyrmex* are: antennae with nine antennal segments lacking an antennal club, and the tumuliform metathoracic spiracles. This latter character is shared with some other species in the genus (see the newly described species), but not all of them. Moreover, the petiole of *Brachymyrmex pilipes* and *Brachymyrmex micromegas* is erect, similar to the state observed in the closely related genus *Myrmelachista* and in contrast to the anteriorly inclined petiole of the monomorphic *Brachymyrmex* species. Nevertheless, given that *Brachymyrmex* is currently diagnosed based on the presence of nine antennal segments lacking an antennal club, we conservatively assign the dimorphic species to the genus for now.

The hypotheses presented here are preliminary and need further testing. Both the monophyly of *Brachymyrmex* and the placement of the dimorphic species within the genus are uncertain. To resolve this uncertainty a more thorough revision of the genus is needed, preferably one that combines morphological analysis with molecular systematics. Such a study is currently underway (Ortiz et al., in prep.) and the results should reveal much about the evolutionary history of this poorly understood ant genus.

## Supplementary Material

XML Treatment for Brachymyrmex micromegas

XML Treatment for Brachymyrmex pilipes

XML Treatment for Brachymyrmex brasiliensis

XML Treatment for Brachymyrmex delabiei

XML Treatment for Brachymyrmex feitosai
